# Does the Clock Make the Poison? Circadian Variation in Response to Pesticides

**DOI:** 10.1371/journal.pone.0006469

**Published:** 2009-07-31

**Authors:** Louisa A. Hooven, Katherine A. Sherman, Shawn Butcher, Jadwiga M. Giebultowicz

**Affiliations:** Department of Zoology, Oregon State University, Corvallis, Oregon, United States of America; Dr. Margarete Fischer-Bosch Institute of Clinical Pharmacology, Germany

## Abstract

**Background:**

Circadian clocks govern daily physiological and molecular rhythms, and putative rhythms in expression of xenobiotic metabolizing (XM) genes have been described in both insects and mammals. Such rhythms could have important consequences for outcomes of chemical exposures at different times of day. To determine whether reported XM gene expression rhythms result in functional rhythms, we examined daily profiles of enzyme activity and dose responses to the pesticides propoxur, deltamethrin, fipronil, and malathion.

**Methodology/Principal Findings:**

Published microarray expression data were examined for temporal patterns. Male *Drosophila* were collected for ethoxycoumarin-O-deethylase (ECOD), esterase, glutathione-S-transferase (GST), and, and uridine 5′-diphosphoglucosyltransferase (UGT) enzyme activity assays, or subjected to dose-response tests at four hour intervals throughout the day in both light/dark and constant light conditions. Peak expression of several XM genes cluster in late afternoon. Significant diurnal variation was observed in ECOD and UGT enzyme activity, however, no significant daily variation was observed in esterase or GST activity. Daily profiles of susceptibility to lethality after acute exposure to propoxur and fipronil showed significantly increased resistance in midday, while susceptibility to deltamethrin and malathion varied little. In constant light, which interferes with clock function, the daily variation in susceptibility to propoxur and in ECOD and UGT enzyme activity was depressed.

**Conclusions/Significance:**

Expression and activities of specific XM enzymes fluctuate during the day, and for specific insecticides, the concentration resulting in 50% mortality varies significantly during the day. Time of day of chemical exposure should be an important consideration in experimental design, use of pesticides, and human risk assessment.

## Introduction

It is increasingly evident that daily synchrony between external light/dark cycles and internal circadian rhythms is essential to optimal health. In addition to readily evident sleep/wake cycles, many behaviors, physiological functions, and biochemical processes oscillate in a 24 hour (circadian) cycle. The core clock mechanism involves two interacting molecular feedback loops that are functionally conserved in circadian systems across species, from the fruit fly *Drosophila* [1] to mammals [2]. The roles of circadian clock-controlled molecular rhythms in adapting organisms to the environment are only beginning to be explored.

Sporadic studies have provided evidence that effects of organophosphate, organochlorine, and pyrethroid pesticides in various pest insect species vary with the time of day at which they are applied [3], [4], [5]. Similar reports have been made in mammalian systems [6], [7], [8], [9], [10], [11]. Recent progress describing the molecular basis of the circadian clock has provided an opportunity for mechanistic investigations of the relationships between circadian clocks and chemical exposures.

Although expression levels of detoxifying enzymes are commonly thought of as constant until induced, microarray studies in several model species have suggested that several xenobiotic metabolizing (XM) genes are expressed in daily rhythms. Genome-wide studies of circadian gene expression have revealed rhythms in expression of multiple genes involved in the toxicological response in flies [12] and mammals [13] and a number of reviews suggest that circadian expression of XM genes may have important implications for human toxicology [14], [15], [16].

Humans are inevitably exposed to pesticides in their diet [17] and pesticide exposure remains a global problem [18], [19]. Development of resistance in insects may increase volume and frequency of pesticide use [20], potentially exacerbating the risk of accidental human exposure and release to the environment. Given that basic molecular mechanisms are shared in insects and mammals, a fundamental understanding of the functional significance of circadian rhythms in chemical exposures may facilitate strategies to reduce adverse events in humans, promote control of pest species, and reduce pesticide use.

*Drosophila melanogaster* is the foundational model organism for investigating circadian rhythms. Here we examine published microarray data of temporal profiles in global gene expression to discern temporal expression patterns in XM-related genes. We noted significant daily fluctuations in the expression of several XM genes and investigated whether these data correspond to functional rhythms in enzymatic activity reflective of xenobiotic metabolism. To systematically investigate whether these rhythms have functional consequences, we profiled the diurnal response to acute exposure to representatives of four classes of pesticides, including propoxur (carbamate), fipronil (phenylpyrazole), malathion (organophosphate), and deltamethrin (pyrethroid).

## Results

### Peak Expression of XM Genes Appears to Cluster in time

**Figure 1:**
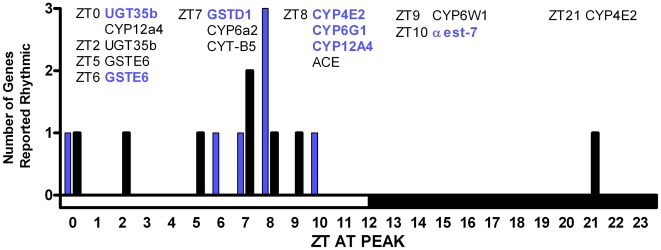
Peak expression times of rhythmically expressed genes implicated in pesticide metabolism and resistance. A list of genes with established or putative cytochrome P450, P450 redox partners, esterase, GST, and UGT function were derived from the literature and Flybase and cross referenced with data from microarray studies [23], [24], [25], [26], [27]. Peak time of expression (x axis) is plotted against frequency of rhythmic genes reported. Solid bars and regular text are from studies of fly head. Blue bars and bold text are from fly body.

Several microarray studies have examined circadian rhythms in global gene expression in *Drosophila* heads and bodies. In general, these studies identified subsets of rhythmically expressed genes with only partial overlap between different reports [21]. Importantly, among genes that have been estimated to be expressed rhythmically in *Drosophila*, rhythms in XM genes were noted in all reports [22]. We assembled rosters of genes by cytochrome P450, esterase, GST, or UGT function, and looked for these genes among rhythmically expressed genes from five reports [23], [24], [25], [26], [27]. We then chose a subset that was linked to pesticide metabolism for further analysis. Figure 1 illustrates peak expression times for genes with reported or suspected involvement in insecticide metabolism and resistance. Of approximately 90 P450s in *Drosophila*, rhythmic expression of 24 genes (27%) has been reported in at least one study. In the literature, at least eight P450s have been implicated in pesticide resistance. Of these, five (63%) are reported rhythmic (Table S1). Of genes implicated in pesticide metabolism and resistance, reported peaks in expression cluster in the day time, particularly late afternoon (Figure 1), and cluster within several hours of each other when reported in more than one study. This is particularly striking in the body: The expression of two GSTs, and three P450s oscillate in phase peaking at ZT6-8. When all P450s and redox partners, esterases, GSTs and UGTs are included in addition to those implicated in pesticide metabolism, a second group of genes peaked between late night and early morning (Figure S1).

### Daily Rhythms in XM Enzyme Activity

**Figure 2:**
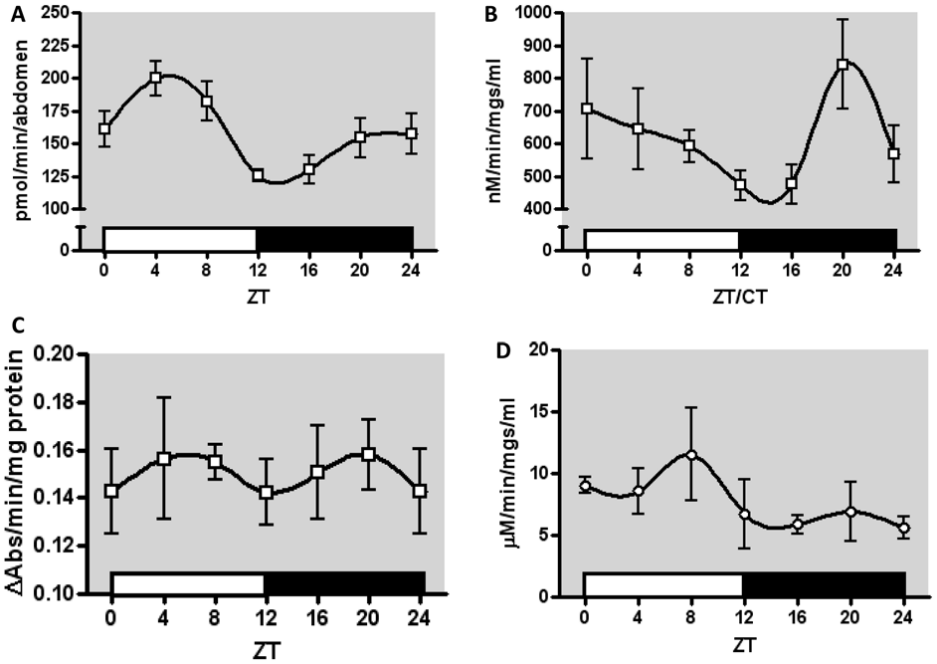
Diurnal variation in enzyme activity in LD conditions. (A) The diurnal profile of ECOD activity showed a significant difference (p = 0.013) in mean activity between the peak at ZT4 and the minimum at ZT12, a difference of 75 pmol 7HC/min/abdomen. (B) The diurnal profile of UGT activity showed a significant difference in means (p = 0.03). A peak in this activity was observed at ZT20 which was significantly different that at ZT16 (p = 0.05), a nearly two-fold difference of 366 nM 4MU/min/mg/ml. (C) The diurnal profile of esterase activity did not show any significant difference between means. (D) The diurnal profile of GST activity did not reveal any significant differences between means. All data are means of 3 biological replicates, with error bars representing SEM.

To examine whether reported expression rhythms in xenobiotic metabolizing genes lead to functional rhythms in enzyme activity, cytochrome P450, esterase, GST, and UGT enzyme activity were assayed (Figure 2). This experiment revealed a significant daily rhythm in P450 activity in unchallenged males. ECOD activity was significantly higher in mid-day than during early night: the mean value at ZT4 was 160% of the value observed at ZT12 (Figure 2A). UGT activity was also significantly rhythmic with nearly two-fold difference between peak enzyme activity at ZT20, and minimum activity at ZT16 (Figure 2B). No significant daily rhythm was observed in the daily profile of esterase activity (Figure 2C) or GST activity (Figure 2D).

### Daily Rhythms in Pesticide Susceptibility to Lethality

**Figure 3:**
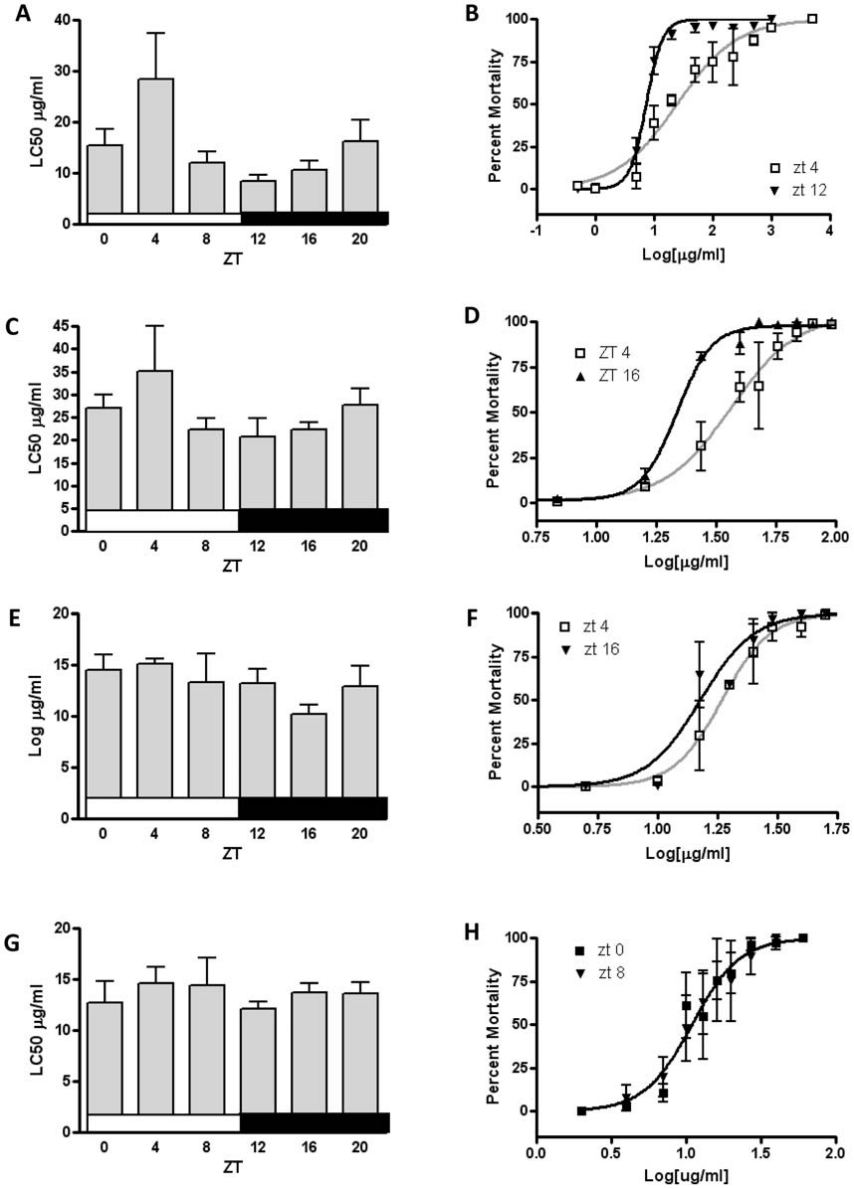
Daily susceptibility profiles from acute exposure to pesticides in LD conditions. (A) LC50s from propoxur exposures throughout the day to concentrations ranging from 0.5 to 1000 µg/ml. (B) LC50 derived from dose response to propoxur at ZT4 (24.6 µg/ml) and ZT12 (7.3 µg/ml) are significantly different [F(2,59)  = 38.74, p<0.0001]. (C) LC50s from fipronil exposures throughout the day (1–300 µg/ml). (D) LC50 from dose response to fipronil at ZT4 (36.5 µg/ml) and ZT16 (21.6 µg/ml) are significantly different [F(4,52)  = 13.11, p<0.0001]. (E) LC50s from malathion exposure throughout the day (1.5–90 µg/ml). (F) LC50s derived from dose response to malathion at ZT4 (18.5 µg/ml) and ZT16 (15.1 µg/ml) are significantly different [F(2,56)  = 3.576, p = 0.0345]. (G) LC50s from deltamethrin exposures throughout the day (1–75 µg/ml). (H) No statistical difference was observed between deltamethrin treatments at ZT0 and ZT8. Error bars represent 95% confidence levels in panels A, C, E, and G,and SEM in B, D, F, and H.

To determine whether daily rhythms in gene expression and activity have physiological consequences in terms of susceptibility, we acutely exposed flies to propoxur, deltamethrin, fipronil, and malathion at ZT0 (lights-on), ZT4, ZT8, ZT12 (lights-off), ZT16, and ZT20 to generate daily susceptibility profiles for each compound. Flies were exposed to a series of doses of propoxur throughout the day in LD (Figure 3A) and two time points were selected to confirm significant difference between them (Figure 3B). The LC50 for propoxur was greatest at ZT4 (24.6 µg/ml), three-fold greater than the LC50 at the time of greatest sensitivity, ZT12 (7.3 µg/ml). The daily susceptibility profile of fipronil exhibited a similar pattern to propoxur (Figure 3C), with greatest LC50 at ZT4 (36.5 µg/ml), which was nearly two-fold higher than at ZT 12–16 (Figure 3D). The daily susceptibility profile of malathion exhibited a rhythm with modest amplitude but significant difference between maximum LC50 of 18.5 µg/ml at ZT4 and the minimum of 15.1 µg/ml at ZT16 (Figure 3E–F). Little variation was observed in daily susceptibility profiles of deltamethrin (Figure 3G), and upon repetition, no statistical difference was found between ZT0 and ZT8 (Figure 3H), or ZT4 and ZT12 (data not shown). A similar non-rhythmic pattern in daily susceptibility profile was observed for another pyrethroid, permethrin (Figure S2).

### Constant Light Suppresses Rhythms in Enzyme Activity and Susceptibility

**Figure 4:**
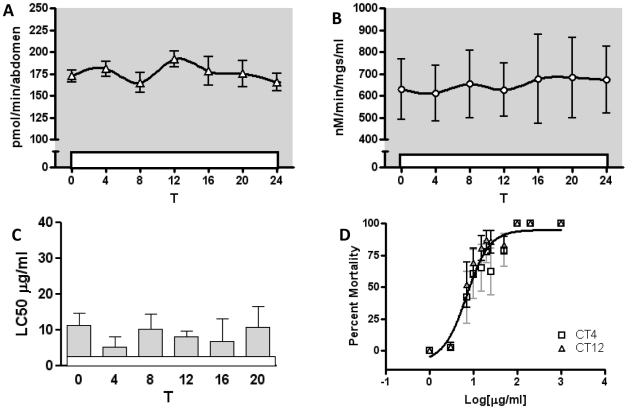
Enzyme activity and daily susceptibility rhythms are abolished in constant light (LL). (A) Daily profile of ECOD activity in LL conditions. (B) Daily profile of UGT activity in LL conditions. (C) LC50s from propoxur exposures throughout the day in LL conditions (1–1000 µg/ml). (D) LC50s derived from dose response at T4 and T12 in LL conditions are not statistically different, and are not statistically different from ZT12 in Figure 3B.

Constant light is known to disrupt the molecular clock mechanism in *Drosophila* [28]. To verify the involvement of the clock mechanism in enzyme activity and susceptibility rhythms, we repeated several experiments in flies held in constant light (LL) for 2 days. Rhythms in ECOD and UGT activity were abolished in LL (Figure 4A–B). Flies subjected to constant light were treated with propoxur at every 4 h at times paralleling those used in LD. The daily rhythm in susceptibility that was observed in flies kept in LD (Fig. 3A–B) was abolished in LL (Fig. 4C), and the LC50 at both T4 and T12 in LL (6.7 µg/ml) was not statistically different from the minimum LC50 at ZT12 in LD (Figure 4B).

## Discussion

Circadian expression of genes related to xenobiotic metabolism (XM) has been observed in both insects and mammals, but no attempts have been made to examine phase distributions for genes with closely related functions. If these rhythms are coordinated, they could have many potential ramifications for responses to chemical exposures. We surveyed circadian variations in mRNA expression of phase I and II enzymes reported by others in microarray profiles of daily gene expression in *Drosophila*. As a category, XM genes were reported as overrepresented among rhythmic genes [22]. Recent meta analyses of previously published microarray expression profiles have added more XM genes [22], [29]. Interestingly, another functional category of P450s related to synthesis of the insect hormone ecdysone, was not reported rhythmic in the microarray studies. While peaks in expression rhythms of all genes were generally distributed across the day in these studies [21], our analysis demonstrated clustering of XM gene expression related to pesticides in the daytime, particularly late afternoon. This implies potential for greater resistance to chemical exposure at this period of the day. While there was little overlap in the specific genes reported as rhythmic in each microarray study [29], our survey of these studies shows remarkable general agreement in the phase of expression of these functionally related genes. All five of the microarray studies examined expression in *Drosophila* head and one in body. Enzyme assays performed in the current work were with abdomens or whole flies. In general, the greatest expression of many XM genes is found in tissues responsible for xenobiotic metabolism, the Malpighian tubules, gut, and crop (http://www.flyatlas.org/) [30] (Table S1). Phase and amplitude of expression may vary between peripheral tissues; therefore, analysis in individual organs is needed to accurately detail organ specific circadian expression patterns of XM genes.

Our study revealed that total activity of some XM-linked enzyme groups show robust diurnal cycling. We found significant daily rhythms in ECOD activity, reflecting mixed function oxidase activity, which has been associated with insecticide resistance and overexpression of cytochrome P450s in *Drosophila* [31]. The greatest ECOD activity was observed between ZT4 and ZT8, consistent with the cluster in reported peaks of P450 mRNA expression. Multiple enzymes and isoforms may contribute to ECOD activity, and the peak in activity we observed may reflect this temporal coordination. Significant daily variation was also observed in UGT activity, with a peak at ZT20. While UGT35b was consistently identified as rhythmic in all five microarray studies, the peak of expression in those studies occurred at ZT2 (±2 h). This difference may reflect a temporal delay between peaks in mRNA and maximum protein activity for UGT35B. Alternatively, it may reflect as yet unidentified rhythms in additional UGT genes. In contrast to P450s and UGTs, GST enzyme activity remained constant throughout the 24h cycle, despite day-time peaks in expression of many GST genes noted previously [22] and confirmed in our study. Similarly, no daily fluctuations in esterase activity were observed in our study.

To examine how these differences in enzyme activity rhythms might influence diurnal physiological response to acute pesticide treatment, commonly used pesticides from four chemical families were selected. Treatment with propoxur, a carbamate, yielded substantial daily variation in mortality. Interestingly, while propoxur is a reversible acetylcholinesterase inhibitor, and malathion (an organophosphate) is an irreversible acetylcholinesterase inhibitor, there was very little daily variation in response to malathion. While acetylcholinesterase itself has been reported to be expressed rhythmically (Figure 1), this result implies that the cause of this difference in daily response does not lie with the target. Rather, given the resemblance in diurnal rhythm of ECOD activity and susceptibility to propoxur, and similar dampening of these rhythms under constant light, it is likely that daily variation in cytochrome P450 activity is responsible for the daily shift in sensitivity to propoxur. Significant daily variation was also observed in response to fipronil, reportedly metabolized by P450s and GSTs [32]. In contrast, we saw no diurnal variation in response to deltamethrin, and thus our results also indicate that this phenomenon is highly dependent on the specific chemical exposure. Interestingly, esterases are thought to play an important role in pyrethroid metabolism [33], and we did not observe significant daily oscillation in esterase activity. Rhythms in response to a single dose of deltamethrin have previously been observed in pine weevil, *Hylobius abietis* [5]; differences between species are possibly due to the substrate specificity of XM genes within each species.

Constant light, which abrogates clock function, depressed rhythms in both enzyme activity and rhythms in susceptibility to propoxur. Interestingly, although the peak in resistance to propoxur occurs in the middle of the light period, a constant light regimen flattened this peak. This indicates that light itself does not mediate the differences in mortality between light and dark periods, but rather suggests that the circadian clock plays a critical role in orchestrating increased day time resistance to some pesticides.

What is the purpose of expression rhythms in the absence of chemical exposure? Induction of XM genes may take many hours to reach full expression levels in both insects and mammals [34], [35]. Daily rhythms in expression of xenobiotic metabolizing enzymes may have evolved to anticipate the intake of plant allelochemicals, mycotoxins, and other compounds ingested during daily feeding rhythms [36], [37]. In flies, the greatest amount of food is eaten in the morning [38], and the current work suggests greatest P450 mRNA expression and activity appears to occur in late afternoon. In mice, feeding peaks at night, and greatest coordinated expression of XM genes is also at night [39]. Temporal compartmentalization of XM activity may also act to degrade daily byproducts of endogenous metabolic processes, prevent inappropriate enzymatic reactions, or regulate production of reactive oxygen species from the microsomal monooxygenase system of which cytochrome P450s are a component [40]. Rhythmic expression of cofactors such as cytochrome P450 reductase, cytochrome b5 (Table S1), heme, and heme oxidase [25], imply that this entire system is under clock coordination.

In mammals, a number of nuclear receptors are known to be expressed rhythmically [41], [42] including the xenosensors aryl hydrocarbon receptor (Ahr), pregnane X receptor (PXR), and constitutive androstane receptor (CAR), which are expressed with similar temporal phase in mouse liver [39]. Disruption of Period gene expression in mice and cultured cells alters the induction of P450s via the AhR in multiple tissues [10], [43], [44]. The *Drosophila* equivalent of AhR, spineless, is not rhythmically expressed and plays a diminished role in response to xenobiotics compared to mammalian species [45].

In mammals, the Clock-Bmal1 complex regulates circadian expression rhythms in DBP and other PARbZip transcription factors, which in turn determine the circadian transcription of CAR [46], [47] and downstream XM genes. Consistent with this data, the degree of inducibility of various mammalian P450 genes varies with circadian time, and this rhythm is abolished by a knock out of the clock-controlled PARbZIP transcription factors DBP, TEF, and HLF [47]. The ability of Pdp1 (the *Drosophila* equivalent of DBP, TEF, and HLF)(Figure S3) to drive the circadian expression of HR96 (the *Drosophila* equivalent of CAR/PXR) is currently under investigation in our laboratory. HR96 is a xenosensor responsible for induction of many xenobiotic enzymes [48]. These reports suggest that the clock machinery is involved in both daily modulation of XM genes and induced expression following xenobiotic exposure. Daily modulation of the xenobiotic response system by the clock would enable the organism to proactively prepare for recurring daily metabolic needs, while reserving readiness to react to greater exposures with classical inductive defenses. The data we present here provide clear evidence that oscillations in expression have functional consequences and that in some cases, the clock together with the dose may make the poison.

Circadian rhythms may impact toxicological endpoints in multiple ways. First, as demonstrated in the current work, circadian rhythms modulate daily fluctuations in enzyme activity, and time of day may have profound influence on the consequences of chemical exposure. While here we focus on metabolism, circadian clocks may also modulate absorption, distribution, excretion, and molecular targets of toxicity, and thus are likely to have broad influence on xenobiotic response. Our study strongly suggests that time of day should be included in insect control strategies and human risk assessment of chemical exposures, including pesticides. Secondly, artificial light, shift work, and jet lag provide examples of how circadian rhythms may be shifted or disrupted, potentially affecting rhythms in XM gene expression and resulting in additive effects between lifestyle and chemical exposure. *Drosophila* is an established model system for many human diseases and conditions including neurodegenerative diseases associated with pesticide exposure [49], [50]. The methods we have established in this work will facilitate future investigation of how circadian coordination modulates xenobiotic metabolism, and how genetic or environmental perturbation of the clock may alter responses to chemical exposure. Finally, *Drosophila* may help to address the open question of how chemical exposures may in turn affect the phase, amplitude, or synchrony of the clock.

## Materials and Methods

### Survey of Temporal Patterns in XM Gene Expression in Drosophila

A roster of cytochrome P450, esterase, GST, or UGT genes was extracted from the literature, Flybase http://flybase.org/ [51], or The Insect P450 Site (http://p450.sophia.inra.fr/index.html). These genes were cross referenced with published microarray data identifying circadian variation in gene expression [23], [24], [25], [26], [27]. Values found in LD were used here, except from the McDonald study, which are from the first day in DD after LD entrainment. We used The Database of Circadian Gene Expression (http://expression.gnf.org/cgi-bin/circadian/index.cgi) to identify several additional rhythmic genes at P≤0.05. All values are used as originally reported in source literature. The daily peak of expression was recorded for significantly rhythmic genes and averaged if reported in multiple studies. The number of genes peaking in expression were plotted against time of day using GraphPad Prism 4.

### Insect Rearing

Canton-S strain of *Drosophila melanogaster* were raised on a cornmeal-molasses-yeast medium at equal densities to ensure uniform size. Sexes were separated under CO_2_ 1–2 days after eclosion, 3–5 days before testing. Stocks were maintained in 12 h light: 12 h dark cycles (LD) at 25°C. Hours after lights-on in LD are expressed as Zeitgeber time (ZT), so that ZT0 is lights-on, and ZT12 is lights-off. Hours are reported as simply time (T) in constant light (LL). For experiments in LL, testing or collections were performed after 48 h of constant light, beginning at T0, parallel with ZT0 in LD.

### Chemicals and Insect Treatment

Insecticides were obtained from ChemService (Westchester, PA) or Sigma-Aldrich (St. Louis MO) and dissolved in acetone. Immediately prior to exposing flies at each time point, the interior of 20 ml glass scintillation vials and caps were coated with 200 µl and 50 µl respectively of desired concentration of pesticide. The vials were rolled on an Old Fashioned Hot Dog Roller (The Helman Group Ltd., Oxnard CA) until evaporation was complete. 3–4 groups of 16 male flies were briefly anesthetized with N_2_ and introduced into the coated vials at ZT0, 4, 8, 12, 16, and 20, an interval used in several of the microarray studies. After 1 hr (±1 min) flies were returned to clean culture tubes with fresh diet. Mortality was recorded 48 or 72 (for fipronil) hours after treatment.

### Enzymatic Assays

4–5 day old male flies were collected at ZT0, 4, 8, 12, 16, 20, and 24, and stored in −80°C until dissection or extraction of sample from whole flies. Protein in each sample was quantified using the BCA method. All assays were performed using a BioTek Synergy 2 plate reader (Winooski, VT).

### 7-ethoxycoumarin-O-deethylase (ECOD) Activity

Mixed function oxidase activity of cytochrome P450s was measured following de Sousa et al [31]. Fly abdomens were dissected and individually placed in the well of a 96-well black microplate (Greiner Bio-One, Frickenhausen, Germany) together with 100 µl of 0.05 M KPO_4_ pH 7.2. After 4 µl of 10 mM 7-ethoxycoumarin in DMSO was added to each well, the plate was centrifuged at 3000 rpm for 2 min, and fluorescence read at (380 nm excitation/450 nm emission). After 4 hr incubation at room temperature, the reaction was stopped using 100 µl GE buffer (1∶1 0.1 mM ph10.4 glycine buffer:ethanol), the plates recentrifuged and re-read. Nine abdomens were assayed for each time point. A standard curve of 7-hydroxycoumarin (7HC) was used to calculate the amount of fluorescent product formed during this time, which was expressed as pmol 7HC/min/abdomen.

### UDP Glucose Transferase (UGT) Activity

UGT activity assay was adapted from Collier et al [52]. Briefly, S9 fraction was prepared from 10 homogenized and sonicated flies by centrifuging for 20 min at 10,000 g in 300 µl 100 mM Tris/HCl pH 7.4 with 5 mM MgCl_2_ and 0.05% BSA. 30 µl of supernatant was added to wells in a black microplate together with 105 µl 200 µM 4-methylumbelliferone (4 MU) and 15 µl of 2 mM uridine 5-diphosphoglucose disodium salt. Decrease in fluorescence of 4 MU by glucosylation was observed at intervals for 3 min at 355 nm excitation and 460 nm emission. A standard curve of 4 MU was used to calculate Vmax as nM 4 MU/min/mg protein.

### Esterase Activity

Three flies were used for each time point, homogenized individually then briefly sonicated in 500 µl 0.05 M KPO_4_ pH 6.8. 90 µl of this solution was transferred to 4 wells of a clear microplate. 90 µl of 0.01 M β-naphthyl acetate was added and the plate incubated at room temperature for 10 min. 90 µl of 1 mg/ml N-(4-amino-2,5-diethoxy phenyl benzamide) in acetone was added, and the plate read at 555 nm. The change in absorbance between samples and controls was reported as change in absorbance/min/mg protein.

### GST Activity

Ten insects from each time point were homogenized then briefly sonicated in 500 µl 100 mM KPO_4_, pH 7.0 with 0.1% Triton X-100. The homogenate was centrifuged at 3,000 g for 5 min, and the supernatant recentrifuged at 10,000 g for 15 min. The supernatant was diluted 1∶5, and 20 µl added to 4 microplate wells containing 170 µl buffer and 20 µl 5 mM GSH. Change in absorbance was observed for 5 min after adding 10 µl of 1 mM 1-chloro-2,4-dinitrobenzidine in acetone. Activity was reported as nM/min/mg protein.

### Statistics

GraphPad Prism 4 was utilized to fit 24 h mortality data to a variable slope sigmoidal dose response curve and calculate LC50s at each time point. An F-test was used to determine whether the dose response curves for the six time points varied significantly. For each compound, the two time points with the largest and smallest LC50s were selected for further analysis. At least two more dose response experiments were repeated at those time points and statistically analyzed as above. For enzyme activities, means of 3 independent experiments are reported. Repeated measures ANOVA and two-tailed t-tests were used to compare peak and trough of enzyme activity.

## Supporting Information

Table S1 Xenobiotic metabolizing genes that have been reported to be involved in pesticide metabolism or resistance and have also been reported to be expressed in a circadian rhythm in Drosophila melanogaster.
(0.13 MB DOC)

Figure S1 Complete histograms of cytochrome P450s and redox partners (top panel), esterases (middle panel), and GSTs and UGTs (bottom panel) found to be rhythmic in microarray studies. Black bars and text are from studies of fly head; hatched blue bars are from fly body. Underlined genes are those related to pesticide metabolism or resistance.
(0.10 MB DOC)

Figure S2 Diurnal susceptibility profile of male flies to permethrin.
(0.04 MB DOC)

Figure S3 Drosophila Clock Mechanism.
(0.27 MB PDF)
