# Supplementary material for: Does the Clock Make the Poison? Circadian Variation in Response to Pesticides
Source: PLoS One. 2009 Jul 31;4(7):e6469. doi: 10.1371/journal.pone.0006469 (PMC2714471; doi:10.1371/journal.pone.0006469)
Supplement: Table S1 — Xenobiotic metabolizing genes that have been reported to be involved in pesticide metabolism or resistance and have also been reported to be expressed in a circadian rhythm in Drosophila melanogaster. (0.13 MB DOC) [file pone.0006469.s001.doc]

| **Name** | **Identifier** | **Time of Peak Expression from Microarray Studies (ZT)** | **Greatest Expression**  **(Fly Atlas)** | **Comments** |
| --- | --- | --- | --- | --- |
| **CYP4E2** | [FBgn0014469](http://flybase.org/reports/FBgn0014469.html)  CG2060 | Head: 21.2 [1]  Body: 8 [2] | Tubule, crop, hindgut, head, male accessory gland, midgut, ovary | [3] |
| **CYP6A2** | [FBgn0000473](http://flybase.org/reports/FBgn0000473.html)  CG9438 | Head: 4 [2,4]  9.2 [1] | Tubule/hindgut | [5] |
| **CYP6G1** | [FBgn0025454](http://flybase.org/reports/FBgn0025454.html)  CG8453 | Body: 8.03 [2] | Tubule/midgut | [6,7] |
| **CYP6W1** | [FBgn0033065](http://flybase.org/reports/FBgn0033065.html)  CG8345 | Head: 8.8 [1] | Carcass, head, thoracicoabdominal ganglion, hindgut, brain | [5] |
| **CYP12A4** | [FBgn0038681](http://flybase.org/reports/FBgn0038681.html)  CG6042 | Head: 0 [2]  Body: 8 [8] | Crop/brain | [9] |
| **Cytochromeb5** | [FBgn0033189](http://flybase.org/reports/FBgn0033189.html)  CG2140 | Head: 6.8 [1] | Body wall/Crop | P450 Redox Partner  [10] |
| **α-Esterase-7** | [FBgn0015575](http://flybase.org/reports/FBgn0015575.html)  CG1112 | Body: 9.7 [2] | Crop, head | AKA Malathion carboxylesterase  [11] |
| **Gst E6** | [FBgn0063494](http://flybase.org/reports/FBgn0063494.html)  CG17530 | Head: 5.1 [2]  Body: 5.9 [2] | Hindgut, tubule | [5] |
| **Gst D1** | [FBgn0001149](http://flybase.org/reports/FBgn0001149.html)  CG10045 | Body: 7.3 [2] | Crop, tubule, whole fly | [12] |
| **Ugt35b** | [FBgn0026314](http://flybase.org/reports/FBgn0026314.html)  CG6649 | Head: 5 [13], 0[2], 2 [1], 2 [14], 0 [15]  Body: 0 [2] | Tubule | [5] |
| **Ace** | [FBgn0000024](http://flybase.org/reports/FBgn0000024.html)  CG17907 | Head: 4 [2] | Thoracicoabdominal ganglion, brain, head | [16] |

1 Ueda HR, Matsumoto A, Kawamura M, Iino M, Tanimura T, et al. (2002) Genome-wide transcriptional orchestration of circadian rhythms in Drosophila. J Biol Chem 277: 14048-14052.

2 Ceriani MF, Hogenesch JB, Yanovsky M, Panda S, Straume M, et al. (2002) Genome-wide expression analysis in Drosophila reveals genes controlling circadian behavior. J Neurosci 22: 9305-9319.

3 Amichot M, Brun A, Cuany A, Helvig C, Salaun L, et al. Expression study of CYP genes in Drosophila strains resistant or sensitive to insecticides. In: Lechner M, editor; 1993 1994; Lisbon, Portugal. Eurotext-John Libbey. pp. 689-692.

4 Ashmore LJ, Sehgal A (2003) A fly's eye view of circadian entrainment. J Biol Rhythms 18: 206-216.

5 Pedra JH, McIntyre LM, Scharf ME, Pittendrigh BR (2004) Genome-wide transcription profile of field- and laboratory-selected dichlorodiphenyltrichloroethane (DDT)-resistant Drosophila. Proc Natl Acad Sci U S A 101: 7034-7039.

6 Daborn PJ, Lumb C, Boey A, Wong W, Ffrench-Constant RH, et al. (2007) Evaluating the insecticide resistance potential of eight Drosophila melanogaster cytochrome P450 genes by transgenic over-expression. Insect Biochem Mol Biol 37: 512-519.

7 Festucci-Buselli RA, Carvalho-Dias AS, de Oliveira-Andrade M, Caixeta-Nunes C, Li HM, et al. (2005) Expression of Cyp6g1 and Cyp12d1 in DDT resistant and susceptible strains of Drosophila melanogaster. Insect Mol Biol 14: 69-77.

8 Hogenesch JB, Su AI (2000) Database of Circadian Gene Expression. Genomics Institute of the Novartis Research Foundation.

9 Bogwitz MR, Chung H, Magoc L, Rigby S, Wong W, et al. (2005) Cyp12a4 confers lufenuron resistance in a natural population of Drosophila melanogaster. Proc Natl Acad Sci U S A 102: 12807-12812.

10 Ranasinghe C, Hobbs AA (1999) Isolation and characterisation of a cytochrome b5 cDNA clone from Helicoverpa armigera (Hubner): possible involvement of cytochrome b5 in cytochrome P450 CYP6B7 activity towards pyrethroids. Insect Biochem Mol Biol 29: 145-151.

11 Wilson RJ, Goodman JL, Strelets VB (2008) FlyBase: integration and improvements to query tools. Nucleic Acids Res 36: D588-593.

12 Tang AH, Tu CP (1994) Biochemical characterization of Drosophila glutathione S-transferases D1 and D21. J Biol Chem 269: 27876-27884.

13 Claridge-Chang A, Wijnen H, Naef F, Boothroyd C, Rajewsky N, et al. (2001) Circadian regulation of gene expression systems in the Drosophila head. Neuron 32: 657-671.

14 McDonald MJ, Rosbash M (2001) Microarray analysis and organization of circadian gene expression in Drosophila. Cell 107: 567-578.

15 Lin Y, Han M, Shimada B, Wang L, Gibler TM, et al. (2002) Influence of the period-dependent circadian clock on diurnal, circadian, and aperiodic gene expression in Drosophila melanogaster. Proc Natl Acad Sci U S A 99: 9562-9567.

16 Menozzi P, Shi MA, Lougarre A, Tang ZH, Fournier D (2004) Mutations of acetylcholinesterase which confer insecticide resistance in Drosophila melanogaster populations. BMC Evol Biol 4: 4.
