# Supplementary material for: Does the Clock Make the Poison? Circadian Variation in Response to Pesticides
Source: PLoS One. 2009 Jul 31;4(7):e6469. doi: 10.1371/journal.pone.0006469 (PMC2714471; doi:10.1371/journal.pone.0006469)
Supplement: Figure S3 — Drosophila Clock Mechanism. (0.27 MB PDF) [file pone.0006469.s004.pdf]

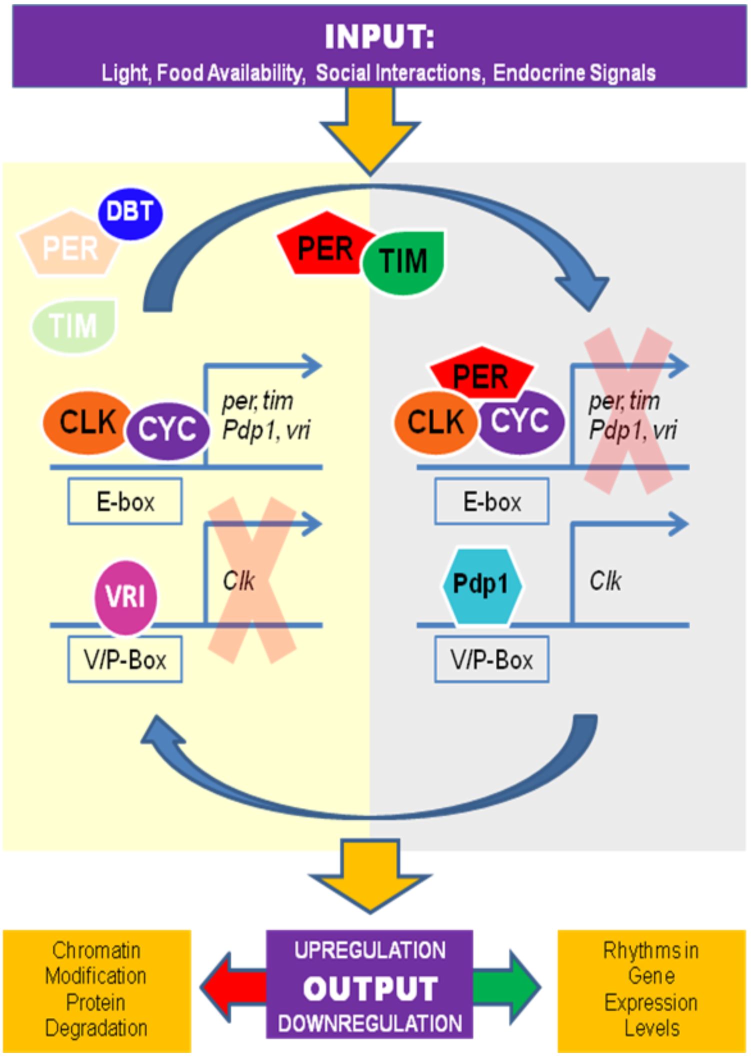

Simplified model of the circadian central clock mechanism. While light is the most potent zeitgeber (timegiver), additional environmental and endogenous conditions may act to reinforce or disrupt the clock . The clock is composed of two interconnected feedback loops [1]. In the daytime (left) DBT initially targets PER protein for degradation via phosphorylation [2], while TIM protein degradation is activated by interaction of CRY (not shown) with daylight [3]. This allows CLK and CYC to activate the transcription of *per*, *tim*, *Pdp1*, and *vri*, while VRI inhibits further transcription of *clk* [4]. 4-6 hours after expression peaks, TIM, which is important for PER stabilization, interacts with PER/DBT and all are translocated to the nucleus with the help of SGG, CK2, and PP2A (not illustrated) [5]. At night (right), inside the nucleus, PER inhibits *per, tim, pdp1* and *vri* transcription by binding to CLK/CYC, while PDP1 activates the transcription of clk [6], or may only function to activate output pathways [7]. In addition to central clock components, many clock-controlled genes are transcribed rhythmically [8], likely due to direct interaction of clock genes with upstream sequences, or through circadian regulation of chromatin remodeling [9], or rhythmic expression of upstream regulators, such as nuclear receptors [10] . Additionally, rhythms in degradative processes may regulate the persistence of proteins and other macromolecules [11]. For a more detailed review of clock mechanism, see Yu et al [12].

1 Hardin PE (2006) Essential and expendable features of the circadian timekeeping mechanism. Curr Opin Neurobiol 16: 686-692.

2 Price JL, Blau J, Rothenfluh A, Abodeely M, Kloss B, et al. (1998) double-time is a novel Drosophila clock gene that regulates PERIOD protein accumulation. Cell 94: 83-95.

3 Lin FJ, Song W, Meyer-Bernstein E, Naidoo N, Sehgal A (2001) Photic signaling by cryptochrome in the Drosophila circadian system. Mol Cell Biol 21: 7287-7294.

4 Hardin PE (2004) Transcription regulation within the circadian clock: the E-box and beyond. J Biol Rhythms 19: 348-360.

5 Harms E, Kivimae S, Young MW, Saez L (2004) Posttranscriptional and posttranslational regulation of clock genes. J Biol Rhythms 19: 361-373.

6 Cyran SA, Buchsbaum AM, Reddy KL, Lin MC, Glossop NR, et al. (2003) vrille, Pdp1, and dClock form a second feedback loop in the Drosophila circadian clock. Cell 112: 329-341.

7 Benito J, Zheng H, Hardin PE (2007) PDP1epsilon functions downstream of the circadian oscillator to mediate behavioral rhythms. Journal of Neuroscience 27: 2539-2547.

8 Wijnen H, Naef F, Boothroyd C, Claridge-Chang A, Young MW (2006) Control of daily transcript oscillations in Drosophila by light and the circadian clock. PLoS Genet 2: e39.

9 Grimaldi B, Nakahata Y, Kaluzova M, Masubuchi S, Sassone-Corsi P (2009) Chromatin remodeling, metabolism and circadian clocks: the interplay of CLOCK and SIRT1. Int J Biochem Cell Biol 41: 81-86.

10 Yang X, Downes M, Yu RT, Bookout AL, He W, et al. (2006) Nuclear receptor expression links the circadian clock to metabolism. Cell 126: 801-810.

11 Ho MS, Ou C, Chan YR, Chien CT, Pi H (2008) The utility F-box for protein destruction. Cellular and Molecular Life Sciences 65: 1977-2000.

12 Yu W, Hardin PE (2006) Circadian oscillators of Drosophila and mammals. J Cell Sci 119: 4793-4795.
