# Supplementary material for: Does the Clock Make the Poison? Circadian Variation in Response to Pesticides
Source: PLoS One. 2009 Jul 31;4(7):e6469. doi: 10.1371/journal.pone.0006469 (PMC2714471; doi:10.1371/journal.pone.0006469)
Supplement: Supplementary file 3 [file pone.0006469.s002.doc]

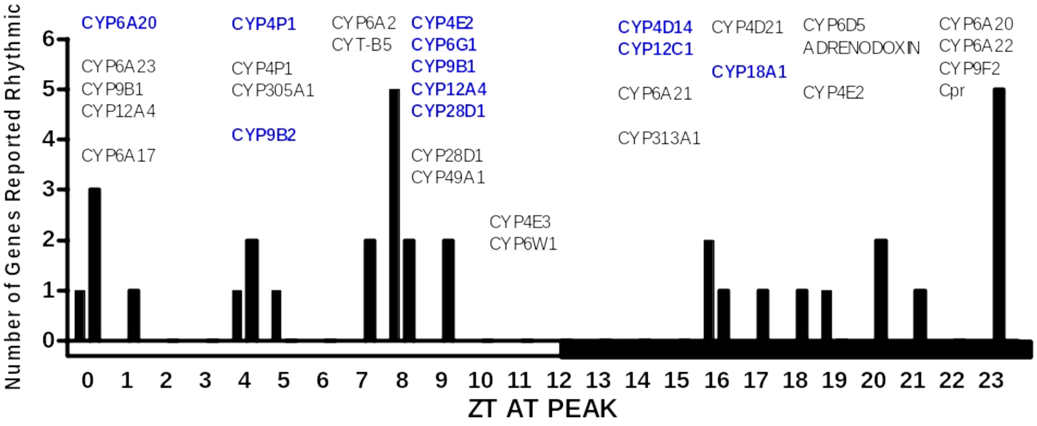
Number of Genes Reported Rhythmic
ZT AT PEAK
CYP6A20
CYP6A23
CYP9B1
CYP12A4
CYP6A17
CYP4P1
CYP4P1
CYP305A1
CYP9B2
CYP6A2
CYT-B5
CYP4E2
CYP6G1
CYP9B1
CYP12A4
CYP28D1
CYP28D1
CYP49A1
CYP4E3
CYP6W1
CYP4D14
CYP12C1
CYP6A21
CYP313A1
CYP4D21
CYP18A1
CYP6D5
ADRENODOXIN
CYP4E2
CYP6A20
CYP6A22
CYP9F2
Cpr

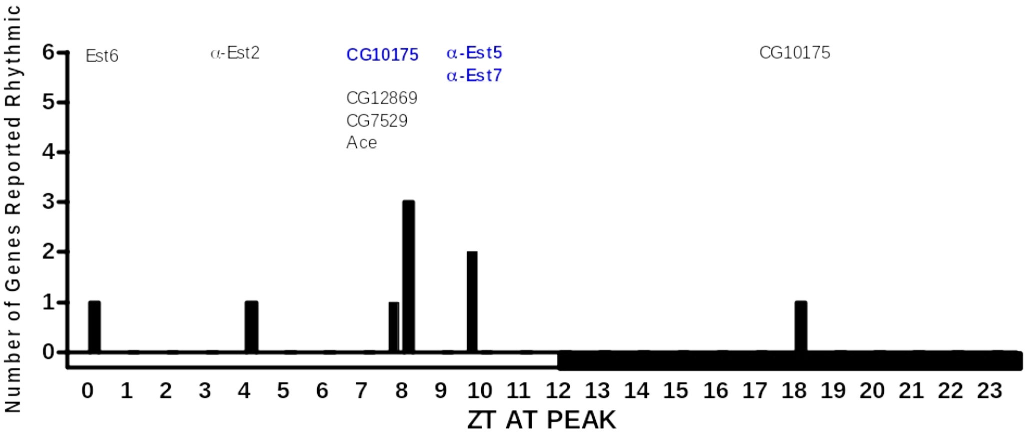
Number of Genes Reported Rhythmic
ZT AT PEAK
Est6
α-Est2
CG10175
CG12869
CG7529
Ace
α-Est5
α-Est7
CG10175

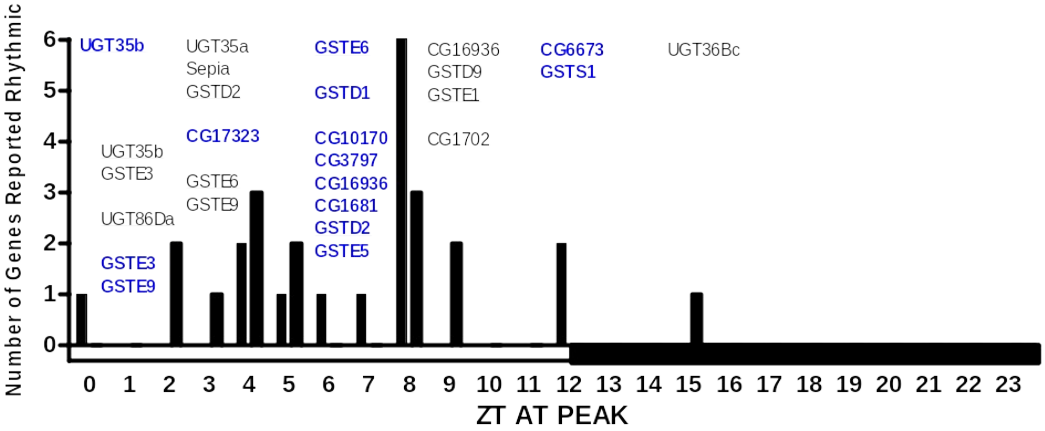
Number of Genes Reported Rhythmic
ZT AT PEAK
UGT35b
UGT35b
GSTE3
UGT86Da
GSTE3
GSTE9
UGT35a
Sepia
GSTD2
CG17323
GSTE6
GSTE9
GSTE6
GSTD1
CG10170
CG3797
CG16936
CG1681
GSTD2
GSTE5
CG16936
GSTD9
GSTE1
CG1702
CG6673
GSTS1
UGT36Bc
