# Supplementary material for: Does the Clock Make the Poison? Circadian Variation in Response to Pesticides
Source: PLoS One. 2009 Jul 31;4(7):e6469. doi: 10.1371/journal.pone.0006469 (PMC2714471; doi:10.1371/journal.pone.0006469)
Supplement: Supplementary file 4 [file pone.0006469.s003.doc]

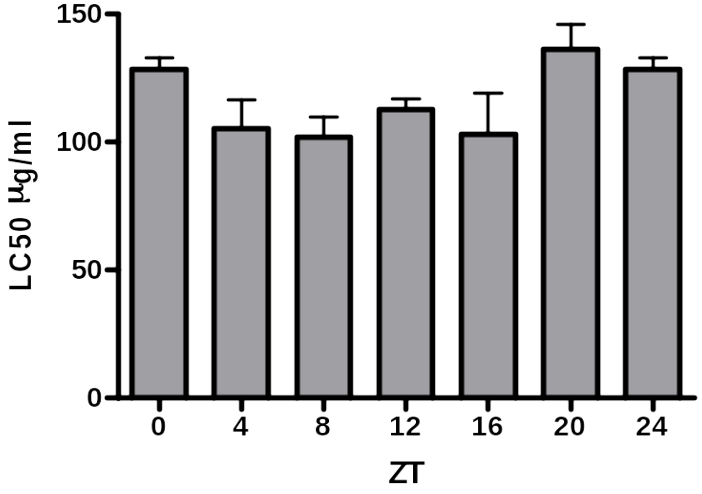

LC50 μg/ml
150
100
50
0
0
4
8
12
16
20
24
ZT
